# Desert Tortoise (*Gopherus agassizii*) Dietary Specialization Decreases across a Precipitation Gradient

**DOI:** 10.1371/journal.pone.0066505

**Published:** 2013-06-28

**Authors:** Ian W. Murray, Blair O. Wolf

**Affiliations:** 1 Department of Biology, University of New Mexico, Albuquerque, New Mexico, United States of America; 2 Brain Function Research Group, School of Physiology, Faculty of Health Sciences, University of the Witwatersrand, Parktown, South Africa; University of Western Ontario, Canada

## Abstract

We studied the plant resource use between and within populations of desert tortoise (*Gopherus agassizii*) across a precipitation gradient in the Sonoran Desert of Arizona. The carbon and nitrogen stable isotope values in animal tissues are a reflection of the carbon and nitrogen isotope values in diet, and consequently represent a powerful tool to study animal feeding ecology. We measured the δ^13^C and δ^15^N values in the growth rings on the shells of tortoises in different populations to characterize dietary specialization and track tortoise use of isotopically distinct C_4_/CAM versus C_3_ plant resources. Plants using C_3_ photosynthesis are generally more nutritious than C_4_ plants and these trait differences can have important growth and fitness consequences for consumers. We found that dietary specialization decreases in successively drier and less vegetated sites, and that broader population niche widths are accompanied by an increase in the dietary variability between individuals. Our results highlight how individual consumer plant resource use is bounded under a varying regime of precipitation and plant productivity, lending insight into how intra-individual dietary specialization varies over a spatial scale of environmental variability.

## Introduction

G. E. Hutchinson conceptualized the idea that the species niche described the multi-dimensional continuum of abiotic and biotic requirements, including resource use, characterizing a species [1]. Following, this resource use space can be narrow in a dietary specialist, or wide in a generalist species [2]. However, it has become increasingly clear that significant levels of variation in resource use may occur between individual consumers within a population [3]–[5]. Because individuals vary in their degree of dietary specialization, a single population can contain both specialists and generalists [6]. Specialists consistently use a narrow subset of available resources, while generalists utilize a greater array of the available resources [2]. This inter-individual variability in diet selection may be due to the unique requirements or phenotypes of different age classes or sexes [7]–[9]. However, even individuals of the same sex and similar age can show differing levels of resource specialization [3], [4]. This variability in individual dietary niche width may importantly influence fitness differences among individuals through such factors as differences in predation pressures or competition [10]–[14]. Differences in resource availability, or ‘ecological opportunity’ [5] among habitats is one factor known to influence the level of individual dietary specialization between populations [14], [15]. The desert tortoise (*Gopherus agassizii*, Cooper 1863) is a long-lived herbivorous reptile that occurs over a wide range of arid habitats in the Mojave and Sonoran Deserts of North America, as well as parts of Sinaloa, Mexico. This species, thus presents an ideal opportunity to examine how dietary specialization among populations varies across a gradient of habitats with differing resource availabilities. In the Sonoran Desert of Arizona, U.S.A., the desert tortoise is typically restricted to isolated, xeric mountain ranges within the Arizona Upland subdivision floristic community [16]. This hot, water-limited environment produces significant physiological challenges for animals inhabiting this region, which include limited and highly variable plant productivity.

How then does the desert tortoise take full advantage of such a highly variable resource environment to maximize survival and growth? Foraging observations and scat analyses of desert tortoises have shown that they feed on a wide variety of grasses, forbs, and shrubs [17]–[19]. Individuals within a population frequently, however, specialize on a small subset of the available plant resources [19]. Although these studies provide significant insight into desert tortoise feeding patterns and nutrition over short periods of time, they still provide only a limited view of the trophic niche space of an animal that can live in excess of sixty years [20], [21].

In this study, we examine the lifetime dietary history of tortoise populations by using the information embedded in the keratinized scute rings on the tortoise’s shell. Because tortoises grow via the sequential addition of distinctly marked growth rings in a fashion similar to tree rings, a cross-section of an individual’s growth rings contains dietary information recorded over its lifespan. We use individual growth rings to quantify the use of specific resource compartments by tortoises, such as C_4_ grasses and CAM plants versus C_3_ forbs and shrubs, to define the lifetime dietary niche breadth of individuals and populations. Quantifying the use of these specific resource compartments is possible because plant photosynthetic groups, (C_3_, C_4_, CAM), often have broadly differing tissue carbon isotope ratios [22]. Because “you are what you eat,” and animals are isotopically linked to their diets, measurements of the δ^13^C of consumer tissues allows for direct estimates of resource use.

We focus on these specific resource compartments to define dietary specialization for several reasons. First, the productivity of these plant functional groups is driven by distinct precipitation pulses (e.g., summer versus winter). Summer rains contribute approximately ½ of total precipitation and primarily drive C_4_ and CAM plant productivity [23], [24]. Winter rains contribute the other ½, and drive C_3_ plant productivity. Precipitation during both seasons decreases markedly from east to west and is also less reliable and more variable in the west [23]. Secondly, and of critical importance to the desert tortoise is the observation that these plant photosynthetic groups differ significantly in their nutritional quality. Tortoises in the Sonoran Desert forage among an annually variable selection of C_3_ forbs, C_3_ shrubs, C_4_ grasses, and succulent CAM plants such as cacti. C_3_ grasses, C_4_ forbs, and cacti are relatively insignificant dietary components on most sites studied in the Sonoran Desert [25]. The majority of a Sonoran Desert tortoise’s diet is made up of C_4_ grasses, C_3_ mallows, and C_3_ desert vine [25]. Due to differences in structural anatomy, C_3_ plant tissues are generally more digestible and yield more energy than C_4_ plant tissues [26], [27]. Additionally, C_3_ forbs have superior nitrogen and water yields relative to grasses [28]–[30]. Thirdly, CAM plants such as cacti, contain large amounts of water, but also have high levels of secondary compounds, which can be toxic or are difficult to digest for some consumers [31]. These plant traits may importantly impact overall rates of energy and nutrient intake because tortoises are only able to process plant material at an exceptionally modest pace [28]. Given these physiological and ecological constraints, it is likely that desert tortoises face considerable challenges in balancing their nitrogen, water, and energy budgets on daily, seasonal and annual bases. As a consequence, foraging decisions can have an important influence on survival, growth and reproduction. Understanding how these decisions vary at different spatial and temporal scales is a primary goal of this study.

The isotopically distinct plant photosynthetic types present in the Sonoran Desert allow the tracking of consumer nutrient use and dietary specialization across variable environments. In the Sonoran Desert ecosystem, stable isotope analyses can provide insight into the degree of individual specialization within the dietary niche [32]. By exploring the variability in plant resource use in tortoise populations currently occurring over a range of habitats with different climatic conditions and resource availabilities, we can better understand the dynamics of individual level resource specialization across a spatially variable gradient.

To characterize the dietary niche of tortoises across a precipitation gradient we use the spatial niche metrics outlined in Layman et al. [33] as well as a Bayesian approach to estimating standard ellipse area to analyze tortoise diet based on growth ring carbon and nitrogen stable isotope ratios [34]. According to Layman et al. [33], the total niche width of a population is determined by the isotopic ‘space’ occupied by individuals on a bi-plot of δ^13^C and δ^15^N ratios, or the total area of the smallest convex polygon that encompasses all of the growth ring carbon and nitrogen ratios. Conceptually, the convex hull total area of a population (TA) accommodates the breadth of resources used by all individuals within a population. We argue that relative to the population TA (TA_p_), the convex hull encompassing each tortoise’s growth rings (TA_i_) is an estimate of individual dietary specialization. Thus, the proportion of TA_p_ that is occupied by the individuals’ TA_i_ (TA_i_/TA_p_) describes the level of specialization within a population. A value of 1 indicates all individuals use all available resources (i.e., generalist), and a value of 0 describes a population of complete specialists, each using a single resource type [2], [3]. Additional metrics such as mean nearest neighbor distance (MNND) and the standard deviation of mean nearest neighbor distance (SDMNND) describe how growth rings from individual tortoises are distributed relative to one another within a population’s dietary niche space [33]. Lower values for these metrics indicate an increased level of spatial overlap (i.e., less dietary divergence) within a tortoise population and provide insights into dietary variability within and between tortoises. Trophic niche width and individual dietary specialization as calculated using the standard ellipse area (SEA) approach of Jackson et al. [34] operates on similar principles, but instead of using convex hull areas, the SEA is used. In this study, a dietary index near one indicates that tortoises are foraging on a mix of C_3_ and C_4_ or CAM plants with keratin values that encompass a more extensive portion of the available (for a given site) spectra of carbon and nitrogen isotope ratios. It follows that individuals in a generalist population can be using either the full extent of available plant resources (i.e., generalists within a generalist population; TA_i_ is broad relative to TA_p_, and there is high overlap between individuals), or using a subset of the available resources with individuals broadly differing in their specialist diet (i.e., specialists within a generalist population; TA_i_ is narrow relative to TA_p_, and there is low overlap among individuals; [35], [36]).

In this study, we address several questions that are particularly relevant given the projected shifts towards a warmer and drier climate that desert organisms like tortoises will experience. Specifically, we ask how tortoise dietary specialization changes across a gradient of increasing aridity and temperature. We hypothesize that as total precipitation decreases and plant resource availability declines, tortoises will adopt a more generalized feeding strategy. Second, we ask whether the degree of tortoise dietary specialization (TA_i_/TA_p_) varies with age class and sex. We suspect that spatial and seasonal differences in activity and ecology between juveniles and adults may increase juvenile tortoise dietary specialization. Thirdly, we estimate the proportion of the total lifetime diet that is harvested from specific ecosystem compartments (C_3_ versus C_4_/CAM plants) across the populations we sample.

## Materials and Methods

### Ethics Statement

All procedures were approved by the Animal Care and Use Committee of the University of New Mexico (09-100244-MCC), and tortoise research on public lands was conducted under permit SAGU-2007-SCI-007 (Saguaro National Park) and Arizona Game and Fish Department scientific collecting permit SP594732.

### Study Area

**Figure 1:**
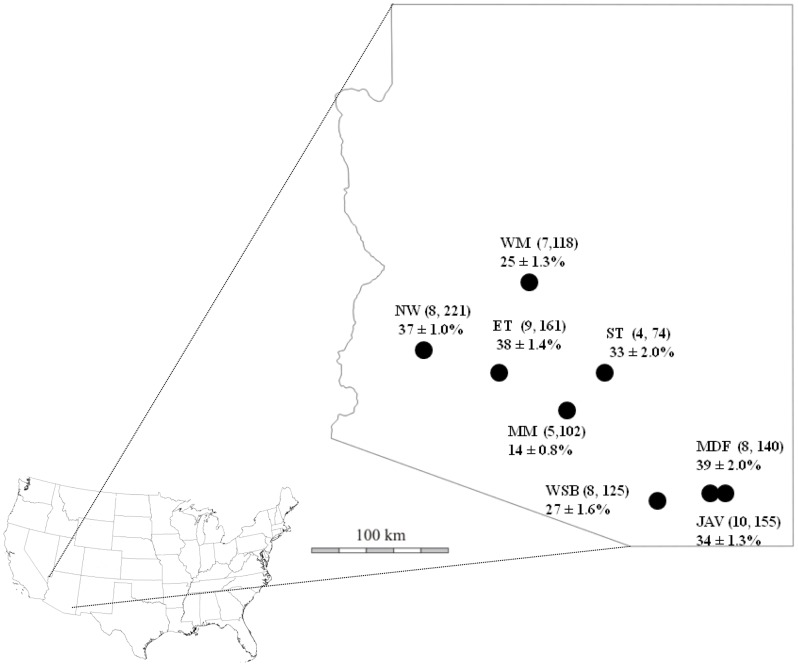
Tortoise sampling sites. Distribution of desert tortoise sampling sites in the Sonoran Desert of Arizona (black dots), followed by the number of tortoises sampled from each sampling site and the total number of scute growth rings analyzed (n,n) for stable isotope analyses per site. Mean population (± SE) utilization of C_4_/CAM plant resources is presented on the map. (WM = Wickenburg Mts.; NW = New Water Mts.; ET = Eagletail Mts.; ST = San Tan Mts.; MM = Maricopa Mts.; WSB = West Silverbell Mts.; MDF = Mother’s Day Fire (Rincon Mts.); JAV = Javelina site (Rincon Mts.). (Map used with permission from http://www.world-geographics.com/.).

We chose eight locations in the Sonoran Desert of Arizona that represent a gradient of increasing aridity and temperature (Figure 1, Table 1). Each site was approximately 2.6 km^2^. Precipitation varied by more than two-fold across the gradient (Table 1). Two sites situated within 5 km of each other (JAV and MDF) shared similar vegetation communities but differed in that one of them had experienced a fire in May of 1994. Insights into how this fire may have affected tortoise plant resource use will be described elsewhere.

**Table 1:** Precipitation metrics by site.

Site*	Annual rainfall (mm)	Proportion Winterrainfall	CV** annual rainfall	CV summer rainfall	CV winter rainfall
**JAV**	327	0.42	27.1	35.1	47.9
**MDF**	326	0.42	26.9	35.1	47.9
**WSB**	252	0.47	29.7	37.0	49.0
**ST**	234	0.56	34.8	47.1	52.2
**MM**	199	0.55	38.3	49.1	56.6
**ET**	176	0.57	41.0	47.6	63.7
**NW**	158	0.55	41.1	50.3	64.9
**WM**	406	0.57	38.2	41.7	61.7

* WM = Wickenburg Mts.; NW = New Water Mts.; ET = Eagletail Mts.; ST = San Tan Mts.; MM = Maricopa Mts.; WSB = West Silverbell Mts.; MDF = Mother’s Day Fire (Rincon Mts.); JAV = Javelina site (Rincon Mts.).

** CV = coefficient of variation.

### Tortoises and Tortoise Tissue Collection

Tortoise shells grow by the peripheral addition of keratinized growth rings on top of the simultaneously growing bony tissue of the carapace and plastron. These rings remain distinct for many years after deposition. The keratin in tortoise growth rings is relatively inert, and records the stable isotope values of the plants eaten during the time of development [37]. Consequently, we can examine plant resource use through the animal’s lifetime within and among tortoise populations.

We sampled a growth ring series from 53 wild desert tortoises between 2007 and 2010. We supplemented these data with the shells of six recently deceased tortoises that we opportunistically salvaged from three of the sites. We obtained scute ring series (strips) by using a razor saw (Revell 88–6964; Elk Grove Village, IL, USA) to lift thin cross-sections (∼15 mm wide) from the 2^nd^ or 3^rd^ costal scutes of tortoises immediately after encountering them in the field. We carefully selected the sampling site for these keratinized strips to bisect all of the growth rings starting at the neonatal scute. Keratin is a non-living tissue that adheres to the living bone underneath. Thus, strips included all of the keratin extending from the dorsal surface of the shell to the bony carapace. The entire process took several minutes, and tortoises showed minimal discomfort beyond being physically detained.

### Precipitation Data

We used the PRISM climate mapping system (http://www.prism.oregonstate.edu/) to characterize the precipitation for each site. The UTM coordinate of the southeast corner of each site was entered and the program provided a site-specific precipitation estimate. Data are presented as mean values using precipitation data from 1950 to 2010.

Because peak tortoise activity in the Sonoran Desert occurs during periods of summer precipitation, metrics of mean annual, winter (November through April), and summer (May through October) precipitation for each of the sites were recorded [38].

### Characterization of Plant Resources

We collected tissue from 88 plant species from six of the eight sites between 2009 and 2010. In most cases, we sampled multiple stems, leaves, and flowers from several individual plants of each species. All plant tissues were dried in a drying oven at 55°C (VWR #1390FM; Batavia, IL, USA) and homogenized with a mortar and pestle to create an amalgamation of the sampled plant parts before analyzing the carbon and nitrogen stable isotope ratios in several aliquots (ca. 1.0 mg) of dried plant homogenate for each plant species. We identified plants to the species level and grouped them according to photosynthetic pathway (C_3_, CAM, or C_4_). The small variances within plant photosynthetic type are due to differences in water use efficiencies, and are minimal relative to the differences between photosynthetic pathway types [39].

We characterized the abundance and species composition of plants on all of the sites. Data on annual plant coverage and species identity was collected for each of the eight plots opportunistically between March and October of 2009 and 2010. Between August and October of 2010 we measured the perennial plant cover on all sites. Due to the remote location of the sites and the variable timing of precipitation, the timing of vegetation measurements between sites was not standardized, although plant data on the burned and unburned sites were always collected within the same two day period. We estimated site-specific perennial plant cover and species composition by tallying the species identity and coverage (m) along five 100 m line-intercept transects in tortoise habitat. We calculated annual plant cover and diversity with 20 cm x 50 cm Daubenmire plots placed every 10 m along each of the five transects. Site specific annual net primary productivity (ANPP; g*m^−2^) was estimated using the mean annual precipitation for a site, following established methods used for hot deserts [40].

### Stable Isotope Analyses

Before analysis we scrubbed keratin samples, and removed scute keratin surface contaminants with a 2∶1 chloroform/methanol wash. We separated individual growth rings from each scute sample under a dissecting stereo microscope (Nikon SMZ800; Melville, NY, USA), with a razor blade, as described in [37]. Tortoise growth ring samples and plant tissues were analyzed for carbon (δ^13^C) and nitrogen (δ^15^N) using a continuous flow isotope ratio mass spectrometer (Thermo-Finnigan IRMS Delta Plus; Waltham, MA, USA) connected to a Costech ECS 4010 Elemental Analyzer (Valencia, CA, USA) in the UNM Earth and Planetary Sciences Mass Spectrometry Lab. The precision of these measurements was ±0.1‰ SD based on repeated measurements of internal lab standards. All sample runs included regularly spaced lab standards (soy δ^13^C = −27.2‰ VPDB; δ^15^N = 2.8‰ AIR) that were calibrated against international standards. All values are reported using delta notation (δ) in parts per thousand (‰) as δX = (R_sample_/R_standard_ –1) * 1000. The R_sample_ and R_standard_ represent the ratio of heavy to light isotopes (^13^C/^12^C or ^15^N/^14^N) for the sample and standard.

The diet-to-tissue discrimination (Δ) that occurs during tissue keratin synthesis in desert tortoises was corrected for by subtracting the experimentally determined carbon (0.8‰) and nitrogen (2.55‰) discrimination factors from tortoise growth ring δ^13^C and δ^15^N values [37]. The percent use of C_4_/CAM vs. C_3_ plant resources was estimated using a two-end-point mixing model [41]:
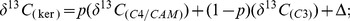
where ‘ker’ is keratin and p is the fraction of C_4_/CAM plant resources assimilated in tortoise scute keratin, and Δ is the keratin carbon discrimination factor (*G. agassizii = *0.8‰).

### Estimates of Dietary Niche Metrics

To estimate tortoise dietary breadth we used the Stable Isotope Analysis in R (SIAR) package to calculate the convex hull area and associated spatial niche metrics for individual and population series of growth ring δ^13^C and δ^15^N ratios [42]. We also used the Bayesian, Stable Isotope Bayesian Ellipses in R (SIBER) package to calculate standard ellipse area (SEA) using growth ring stable isotope data [42], [34]. The SEA is a metric comparable to the convex hull area, but is less sensitive to sample size and takes into account uncertainty within the dataset [34]. We calculated the SEA of individuals (SEA_i_) relative to the SEA of the population (SEA_p_) to estimate dietary specialization (SEA_i_/SEA_p_). We estimate dietary specialization using the δ^13^C and δ^15^N bi-space occupied by individual tortoise growth rings in a population, but we note that nitrogen variability is likely to be due to regional differences in background nitrogen ratios or a reflection of the use of plants that are nitrogen fixers versus those that are not, rather than an indication of different trophic levels in these herbivorous tortoises. Additionally, plant nitrogen isotope values are known to be correlated strongly with site specific soil and precipitation characteristics [43], [44].

## Results

### Sampling sites

All populations sampled occurred on boulder-strewn hillsides and rocky ridges within the Arizona Upland subdivision plant community. The wettest site in the northwest (Wickenburg Mts.; 406 mm) had two and a half times more precipitation relative to the driest site (New Water Mts.; 158 mm). However, the Wickenburg Mts. site may be an outlier due to its significantly cooler air temperatures contributing to especially low tortoise densities there [45]. This, coupled with this site’s high precipitation variability (Table 1), may mean that it is more closely allied to drier tortoise sites in the context of tortoise ‘ecological opportunity’ [5]. Excluding the Wickenburg Mts. site and measuring precipitation on a strict east to west transect we observed two times as much annual precipitation in the Rincon Mountains (327 mm) compared to the New Water Mountains (158 mm). These differences in annual precipitation translated into a two-fold difference in the estimated annual net primary productivity (ANPP; Table 2) potentially available as tortoise forage (JAV; 109 g*m^−2^ vs. NW; 46 g*m^−2^). The coefficient of variation for seasonal summer (May – October) precipitation, a measure of unpredictability, significantly increased as mean annual summer rainfall decreased across sites (y = −7.1x +431.5; df = 7; r^2^ = 0.79; *P* = 0.002). The coefficient of variation for winter and annual precipitation did not significantly increase with decreasing rainfall when the Wickenburg Mts. site was included. When the Wickenburg Mts. site was removed the coefficient of variation significantly increased as both winter and annual rainfall decreased across sites. Estimates of ANPP sharply decrease with increasing annual rainfall coefficient of variation (including WM; y = −2.9x +183.5; r^2^ = 0.28; *P* = 0.2; excluding WM; y = −4.0x +212.5; r^2^ = 0.94; *P* = 0.000).

**Table 2:** Site-specific annual net primary productivity (ANPP) as estimated from [40] and the percent composition of plant functional groups by site.

		Perennial Plant Cover**				Herbaceous Plant Cover					
Site*	ANPP (g*m-2)	% CAM plant cover	% C4 grass cover	% C3 shrub/tree cover	Total Perennial plant cover (m)	% C3 forb cover	% C3 sub-shrub cover	% C3 grass cover	% C4 grass cover	% C4 forb cover	Total herbaceous cover (cm2)***
**JAV**	109.4	0.39	0.0	0.61	158	0.02	0.21	0.0	0.57	0.09	15,810
**MDF**	109.4	0.17	0.02	0.81	114	0.07	0.10	0.0	0.55	0.04	19,410
**WSB**	81.3	0.03	0.0	0.97	103	0.92	0.01	0.0	0.0	0.07	750
**ST**	74.5	0.05	0.0	0.95	76	0.93	0.04	0.0	0.0	0.0	1,410
**MM**	61.2	0.02	0.0	0.98	115	0.96	0.04	0.0	0.0	0.0	19,260
**ET**	52.4	0.0	0.0	1.0	90	0.90	0.03	0.05	0.01	0.0	34,560
**NW**	45.6	0.09	0.0	0.91	21	0.90	0.05	0.0	0.03	0.01	19,500
**WM**	139.0	0.05	0.06	0.87	139	0.03	0.30	0.0	0.25	0.16	1,580

* WM = Wickenburg Mts.; NW = New Water Mts.; ET = Eagletail Mts.; ST = San Tan Mts.; MM = Maricopa Mts.; WSB = West Silverbell Mts.; MDF = Mother’s Day Fire (Rincon Mts.); JAV = Javelina site (Rincon Mts.).

** Perennial plant cover is presented as the sum in meters of canopy cover intercepting line transects, and herbaceous plant cover is presented as the area (cm^2^) occupied of frame transects. Where cumulative percentages do not add to 1.0, the difference is made up of unidentified grass or forb sprouts and mosses.

*** Herbaceous vegetation cover data was pooled across two years.

### Plant Analyses

**Figure 2:**
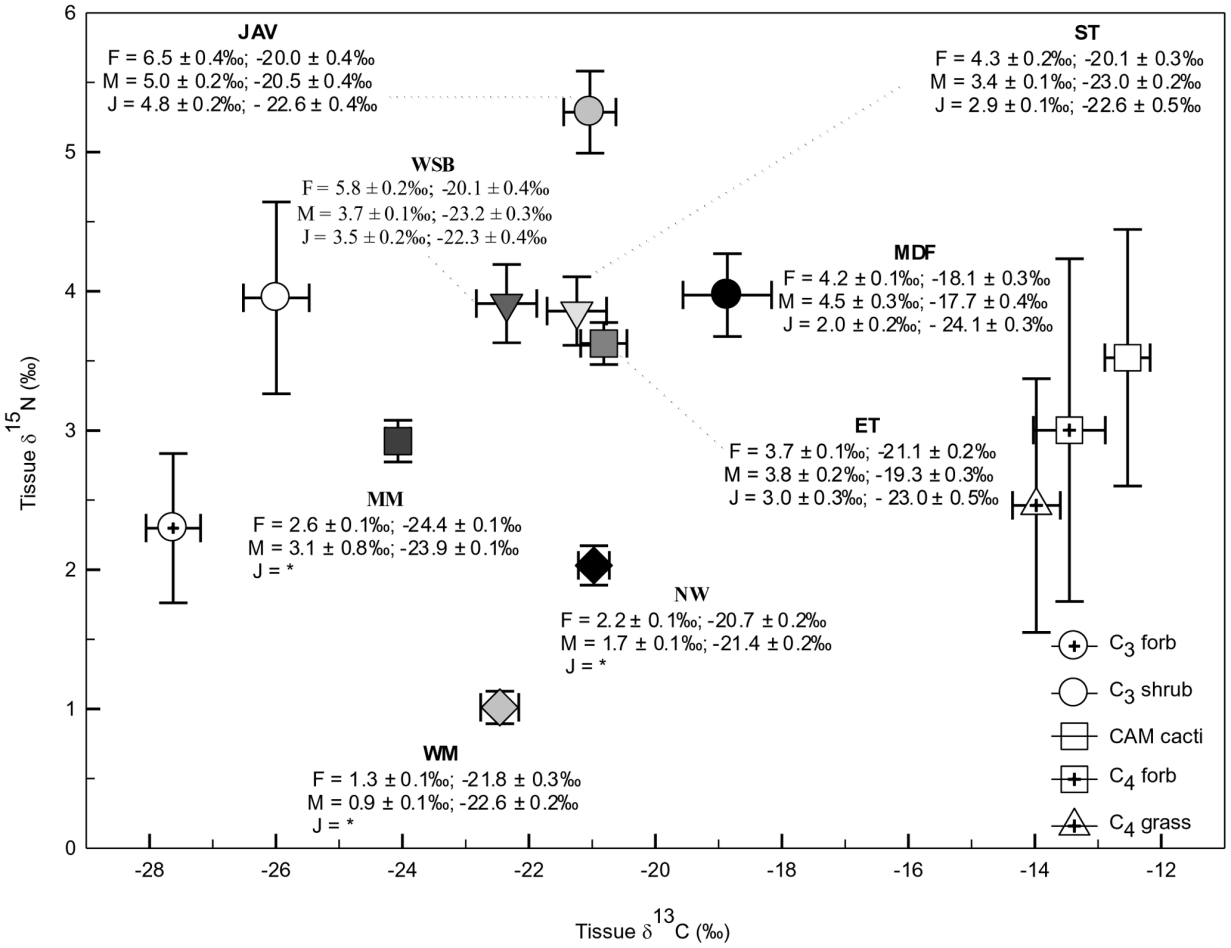
Population specific stable isotope values. Mean desert tortoise growth ring keratin δ^13^C and δ^15^N values plotted for eight populations across Arizona (symbols), as well as mean male (M), female (F), and juvenile (J) growth ring keratin δ^13^C and δ^15^N values by population. Scute growth ring carbon and nitrogen values are plotted relative to mean values (± SE) for plant functional groups (88 species; C_3_ forbs, C_3_ shrubs, C_4_ grasses, C_4_ forbs, CAM cacti) available as plant resources for grazing desert tortoises. Tortoise scute ring δ^13^C (0.8‰) and δ^15^N (2.55‰) values have been adjusted by subtracting the appropriate keratin diet-tissue-discrimination factors in tortoises. (WM = Wickenburg Mts.; NW = New Water Mts.; ET = Eagletail Mts.; ST = San Tan Mts.; MM = Maricopa Mts.; WSB = West Silverbell Mts.; MDF = Mother’s Day Fire (Rincon Mts.); JAV = Javelina site (Rincon Mts.).

Plant functional groups growing in desert tortoise habitat occupied distinct positions in plotted stable isotope space with mean δ^13^C values between −27.6‰ and −12.5‰ VPDB, and mean δ^15^N values ranging from 2.3‰ to 4.0‰ AIR (Figure 2). These data show that desert tortoises select plant resources from among a diverse array of plants with disparate stable isotope values.

Plant community composition changed as the amount and predictability of summer precipitation declined across sites. Across all sites, perennial plant community structure was dominated by C_3_ shrubs and trees (Table 2). Cacti (CAM) always made up a miniscule proportion of plant cover (except the MDF and JAV sites), and CAM plant cover declined precipitously as summer rainfall decreased (y = 1.5x –0.61; df = 7; r^2^ = 0.46; *P* = 0.04). Herbaceous plant cover was dominated by C_3_ forbs on all but the three wettest sites (MDF, JAV, and WM) where C_4_ grasses made up the majority of plant cover (MDF and JAV) or were second only to C_3_ sub-shrubs in their relative importance (WM). C_4_ forbs made up a minimal part of the herbaceous plant biomass across all of the sites.

### Desert Tortoise Trophic Niche Breadth Across an Environmental Gradient

**Figure 3:**
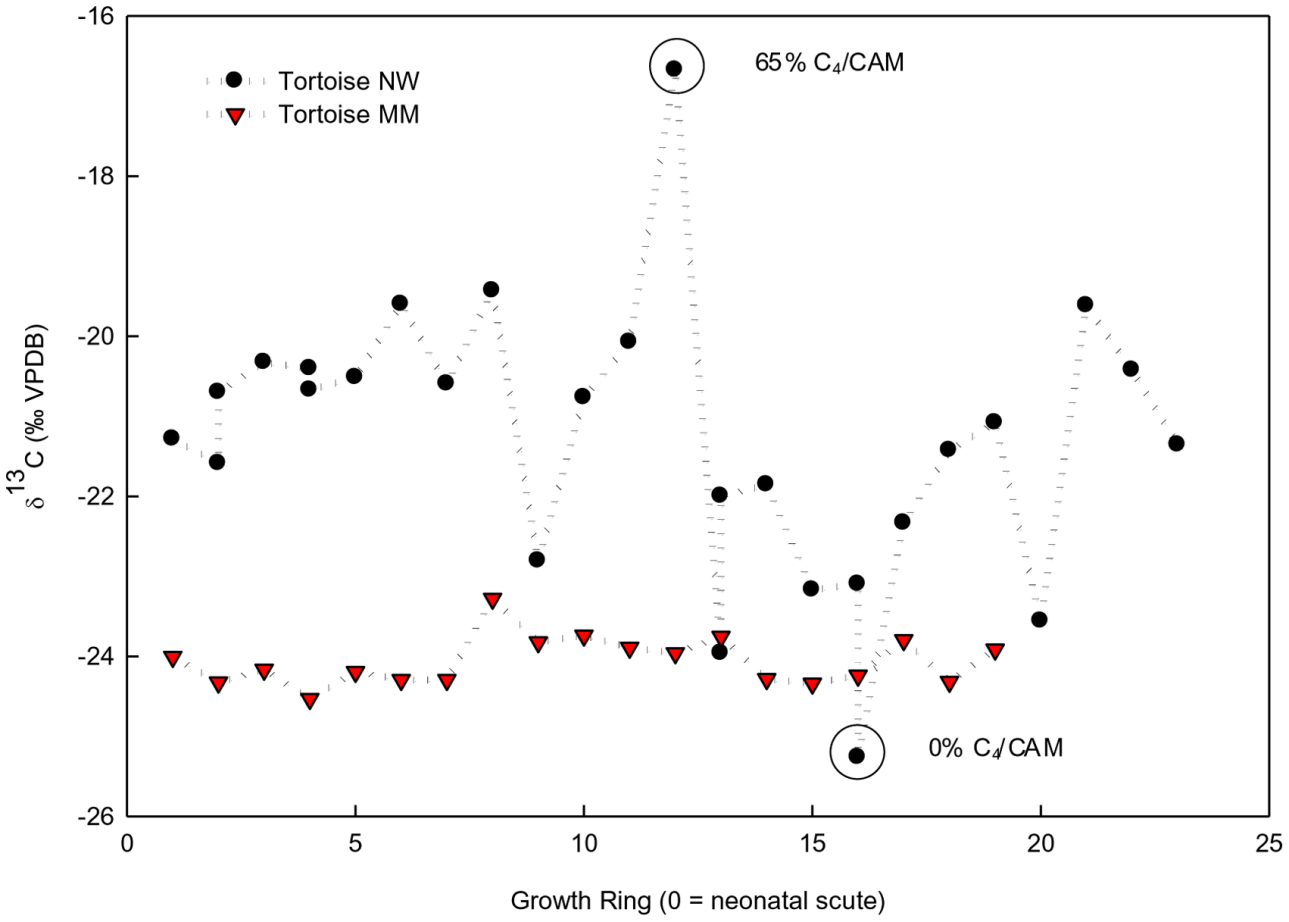
Individual growth ring carbon isotope ratio history. The δ^13^C ratios from a complete sequence of growth rings from an individual tortoise with large, episodic shifts between incorporating C_3_ and C_4_/CAM plant resources (Tortoise NW from the New Water Mts., mean annual precipitation of 158 mm) and an individual tortoise (Tortoise MM from the Maricopa Mts., mean annual precipitation of 199 mm) showing a constant and high reliance on C_3_ plants across its lifetime. Tortoises grow by the successive additions of concentric rings, thus ring 1 would be the ring grown post-hatching, and rings added later in life are numbered consecutively.

**Figure 4:**
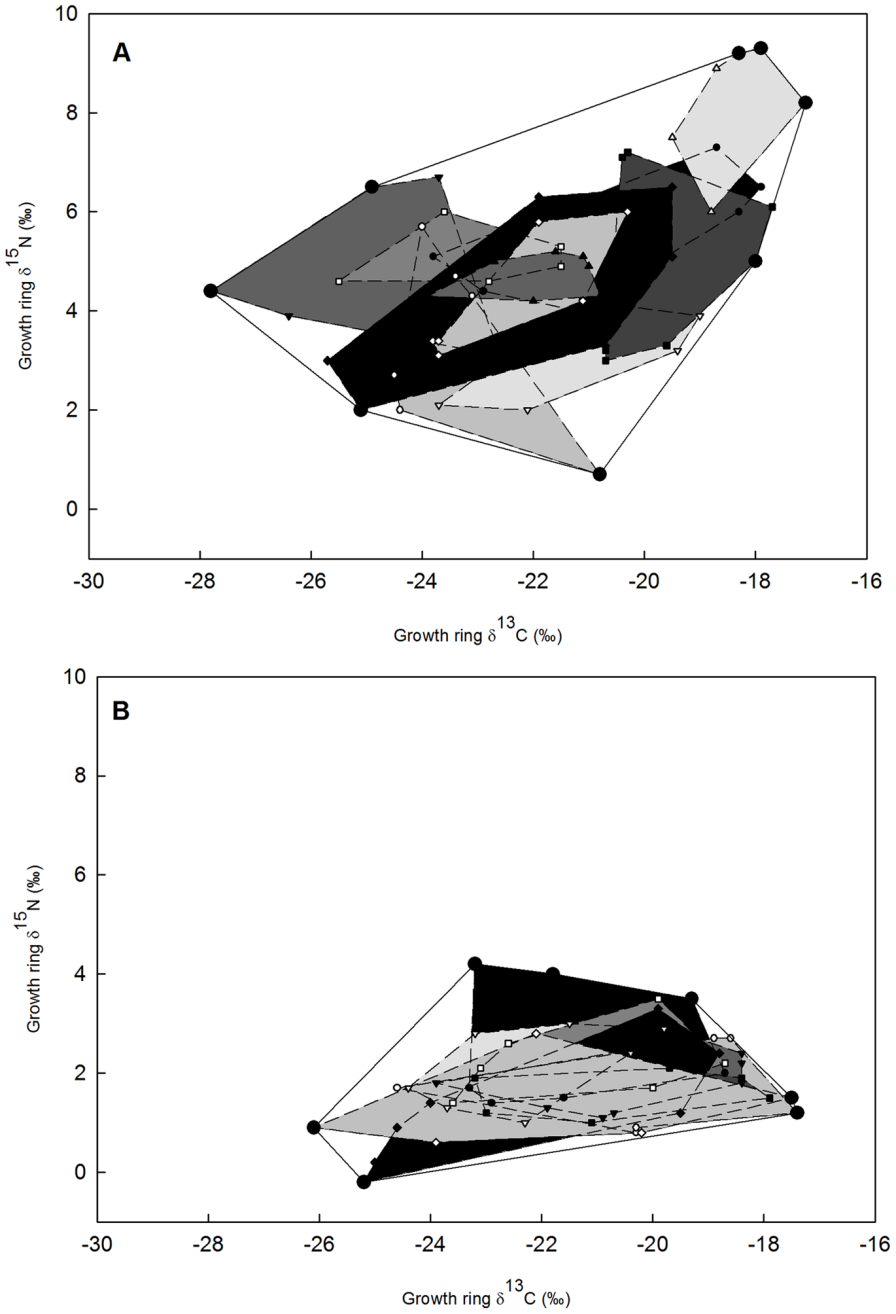
Individual tortoise dietary specialization between sites. Convex hulls for growth ring series from individual tortoises (each polygon represents a single tortoise’s dietary history) plotted relative to the convex hull for all tortoise growth rings for A) the wet Javelina plot and B) the driest plot, the New Water Mts. Desert tortoise dietary specialization decreases across a precipitation gradient based on the area of the convex hull (TA_i_/TA_p_; w/WM: y = −0.001x +0.34; r^2^ = 0.21; w/o WM: y = −0.003x +0.44; r^2^ = 0.67) or based on the standard ellipse area (SEA_i_/SEA_p_; w/WM: y = −0.001x +0.59; r^2^ = 0.25; w/o WM: y = −0.003x +0.72; r^2^ = 0.81). The Wickenburg Mts. (WM) site is an outlier due in part to the colder temperatures there.

Our measurements of the δ^13^C and δ^15^N in 1,096 growth rings from 59 tortoises across eight sites (Table 3) showed that tortoises fed on a mixed diet of plants distributed among the available photosynthetic pathways and functional groups. Moreover, substantial variation was found in the use of plant resources across years for individual tortoises (Figure 3). At one extreme, the diet of the Maricopa Mountains tortoise population was comprised of 86% C_3_ plants compared to 61% C_3_ plants on the Mother’s Day Fire site in the Rincon Mountains (Figure 1). Across an east-west gradient of decreasing precipitation and increasing temperatures the dietary niche of individual tortoises became more generalized (TA_i_/TA_p_ and SEA_i_/SEA_p_ increased; Tables 4 & 5; Figures 4a & 4b). These changes were also accompanied by a decrease in the tortoise population niche width (TA_p_) as precipitation decreased and became more variable (y = −1.45x +82.3; r^2^ = 0.51; df = 7; *P* = 0.029; SEA_p_ showed a similarly significant relationship). Additionally, the population MNND and SDNND of tortoise growth rings increased significantly as precipitation became more variable (MNND; y = −0.013x +0.75; r^2^ = 0.8; df = 7; *P* = 0.002; SDNND; y = −0.014x +0.78; r^2^ = 0.45; df = 7; *P* = 0.04).

**Table 3:** The number (n) of tortoises and scute growth rings sampled for stable isotope analyses for each site.

Site*	N(# rings)	N(# tortoises)	M:F:J**	MCL (mm)***
MM	102	5	3∶2:0	257 (246–267)
WM	118	7	6∶1:0	240 (206–271)
WSB	125	8	2∶1:5	147 (52–228)
ST	74	4	1∶2:1	215 (89–269)
Jav	155	10	5∶2:3	204 (121–259)
NW	221	8	5∶3:0	274 (261–295)
ET	161	9	2∶5:2	228 (80–288)
MDF	140	8	3∶2:3	220 (153–269)

* WM = Wickenburg Mts.; NW = New Water Mts.; ET = Eagletail Mts.; ST = San Tan Mts.; MM = Maricopa Mts.; WSB = West Silverbell Mts.; MDF = Mother’s Day Fire (Rincon Mts.); JAV = Javelina site (Rincon Mts.).

** Males:Females:Juveniles.

*** Midline carapace length.

**Table 4:** Calculated desert tortoise niche metrics based on convex hull areas 1) the range of tortoise growth ring δ^15^N 2) the range of tortoise growth ring δ^13^C 3) MNND – mean nearest neighbor distance for growth rings 4) SDMNND – standard deviation of mean nearest neighbor distance for growth rings 5) Population TA – total area of the population convex hull 6) Mean individual TA_i_ – the mean area of an individual tortoise’s convex hull 7) Mean TA_i_/TA_p_ – mean individual dietary specialization based on the proportion of the population convex hull (TA_p_) occupied by an individuals’ convex hull (TA_i_).

Sex/Site*	δ^15^N range	δ^13^C range	MNND	SDMNND	Population TA	Mean individual TA_i_ (±SE)	Mean TA_i_/TA_p_ (±SE)
**Juvenile (14, 138)** ** **(14,138)** **	8.0	10.6	0.29	0.28	51.7	5.4±1.3	0.13±0.03
**Male (27, 545)**	8.4	13.3	0.19	0.20	75.8	7.7±0.8	0.27±0.03
**Female (18, 391)**	10.8	11.1	0.18	0.17	66.5	7.0±1.0	0.24±0.04
**JAV**	8.6	10.8	0.35	0.30	49.7	6.7±1.2	0.13±0.02
**MDF**	4.9	12.3	0.41	0.41	38.3	5.5±1.1	0.14±0.03
**WSB**	7.5	12.3	0.38	0.50	41.8	9.3±1.7	0.22±0.04
**ST**	5.3	8.4	0.30	0.27	30.7	10.1±3.9	0.33±0.13
**MM**	6.0	5.2	0.18	0.21	16.3	5.2±1.9	0.32±0.12
**ET**	5.5	9.6	0.25	0.19	35.7	6.8±1.7	0.23±0.05
**NW**	4.3	8.7	0.20	0.28	22.9	7.0±0.9	0.31±0.04
**WM**	4.0	8.1	0.21	0.15	21.3	6.1±1.6	0.28±0.08

* JAV – Javelina site (Rincon Mts.), MDF - Mother’s Day Fire site (Rincon Mts.), WSB - West Silverbell Mts., ST - San Tan Mts., MM - Maricopa Mts., ET - Eagletail Mts., NW - New Water Mts., and WM - Wickenburg Mts.

** (number of tortoises, number of growth rings).

**Table 5:** Calculated desert tortoise niche metrics based on standard ellipse areas 1) Population SEA – total area of the population standard ellipse area 2) Mean individual SEA_i_ – the mean of an individual tortoise’s standard ellipse area 3) Mean SEA_i_/SEA_p_ - mean individual dietary specialization based on the proportion of the population standard ellipse area (SEA_p_) occupied by an individuals’ standard ellipse area (SEA_i_).

Sex/Site*	Population SEA_p_	Mean individual SEA_i_ (±SE)	Mean SEA_i_/SEA_p_ (±SE)
**Juvenile (14,138)** **	8.0±0.02	2.6±0.5	0.37±0.07
**Male (27, 545)**	10.9±0.01	2.9±0.3	0.50±0.05
**Rings 1–11 (M; 27, 305)**	10.4±0.02	2.3±0.2	0.41±0.03
**Rings >11 (M; 27, 240)**	11.2±0.02	3.2±0.3	0.58±0.05
**Female (18, 391)**	10.5±0.02	2.6±0.3	0.43±0.05
**Rings 1–11 (F; 18, 203)**	10.9±0.02	2.0±0.1	0.37±0.04
**Rings >11 (F; 18, 188)**	9.8±0.02	2.7±0.4	0.48±0.06
**JAV**	9.1±0.02	3.2±0.6	0.35±0.06
**MDF**	7.0±0.02	2.5±0.5	0.36±0.07
**WSB**	9.1±0.03	4.7±1.1	0.41±0.05
**ST**	6.5±0.02	3.5±1.1	0.54±0.17
**MM**	2.7±0.01	1.8±0.7	0.57±0.18
**ET**	6.4±0.02	2.8±0.5	0.50±0.09
**NW**	4.2±0.01	2.7±0.5	0.56±0.06
**WM**	4.2±0.01	2.1±0.5	0.52±0.12

* JAV – Javelina site (Rincon Mts.), MDF - Mother’s Day Fire site (Rincon Mts.), WSB - West Silverbell Mts., ST - San Tan Mts., MM - Maricopa Mts., ET - Eagletail Mts., NW - New Water Mts., and WM - Wickenburg Mts.

** (number of tortoises, number of growth rings).

### Diet and Tortoise Life Stage and Sex

Adult and juvenile tortoises showed differences in their dietary niche and degree of specialization. We also found that male and female tortoises had different patterns of plant resource use, but similar levels of dietary specialization (Tables 4 & 5). The mean δ^13^C values of scute keratin of female tortoises (n = 391; δ^13^C = −20.9±0.1‰ VPDB) were significantly enriched compared to male δ^13^C values (n = 545; δ^13^C = −22.0±0.1‰ VPDB), which were enriched in ^13^C above juvenile tortoise carbon values (n = 138; δ^13^C = −22.6±0.2‰ VPDB; Tukey’s HSD; *P*<0.05). Juvenile desert tortoise trophic niche (TA_p_) was 32% smaller relative to male tortoise TA_p_ (51.7 vs. 75.8) and 22% smaller than female tortoise TA_p_ (51.7 vs. 66.5; Table 4). Female desert tortoise TA_p_ was 12% smaller relative to male tortoises (66.5 vs. 75.8; Table 4). The standard ellipse area (SEA_p_) for all juvenile tortoises (8.0±0.02) was significantly smaller that for male (10.9±0.01) and female (10.5±0.02) tortoises (Tukey’s HSD; *P*<0.05; Table 5). Female tortoises had only slightly smaller SEA_p_ relative to males, but this difference was statistically significant (two sample t-test; *P* = 0.000; Table 5). Individual juvenile tortoises were almost twice as specialized (TA_i_/TA_p_; 0.13±0.03) compared to individual female and male tortoises (0.24±0.04 and 0.27±0.03, respectively) and this difference was significant (two sample t-test; *P*<0.05; Table 4). However, dietary specialization in juvenile tortoises calculated using standard ellipse areas (SEA_i_/SEA_p_; 0.37±0.07) was not significantly different compared to female and male tortoises (0.43±0.05 and 0.50±0.05, respectively; Table 5).

**Figure 5:**
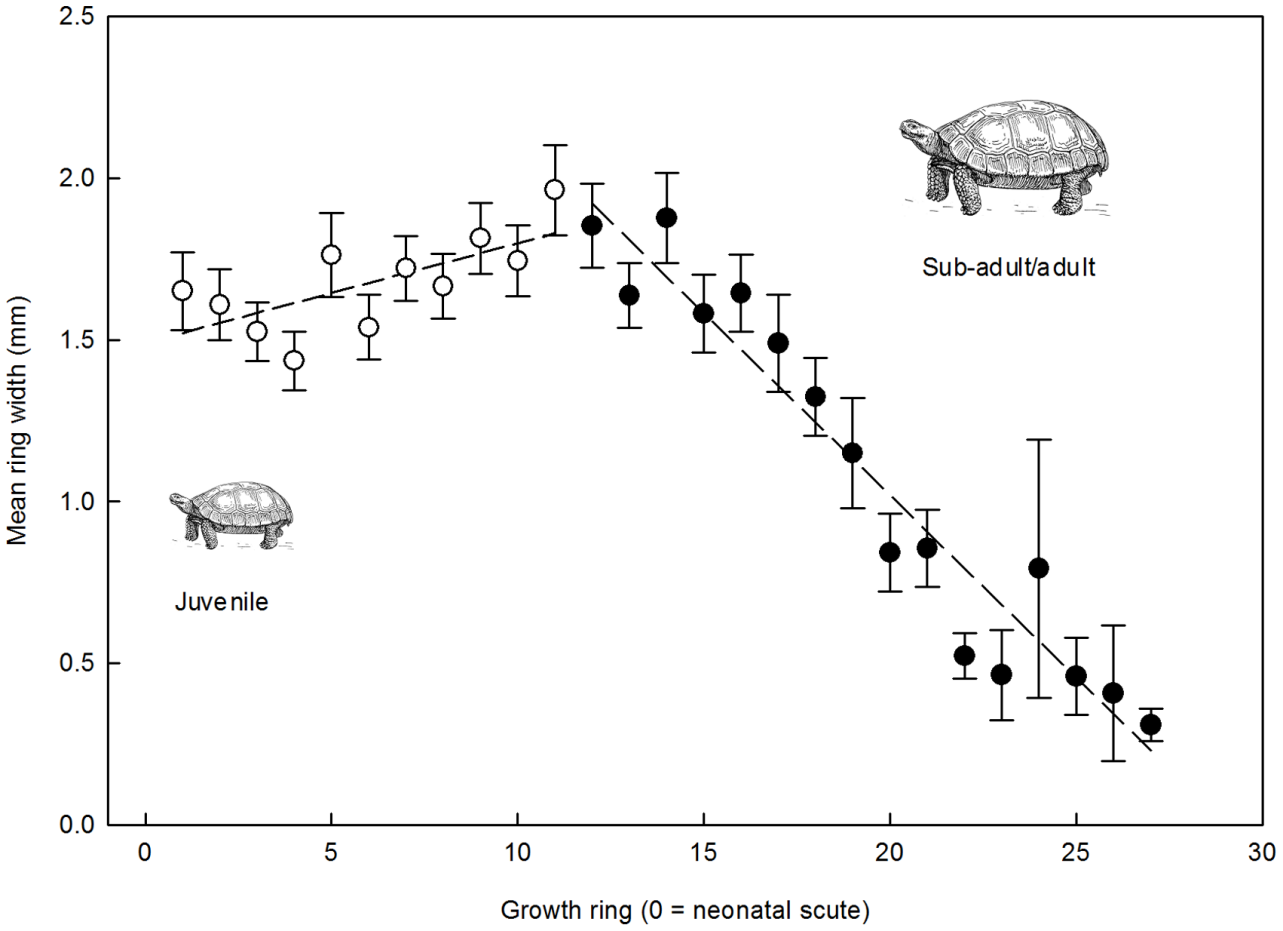
Growth ring width through ontogeny. Pooled desert tortoise growth ring widths, with standard errors, comparing dietary niche metrics on either side of a natural inflection point (segmented regression; y = 1.43+0.05×_1_–0.10×_2_; r^2^ = 0.93) in the growth patterns between juvenile and subadult/adult animals.

We observed a notable change in tortoise growth ring width with age. Mean desert tortoise growth ring widths increased from the first ring grown after hatching, to ring 11, at which point there was a sharp decline in ring width throughout the rest of the growth chronology of the individual (segmented regression; y = 1.43+0.05×_1_–0.10×_2_; r^2^ = 0.93; Figure 5). During the growth period covering the first 11 rings female and male tortoises had more specialized diets (SEA_i_/SEA_p_; males = 0.41±0.03; females = 0.37±0.04) compared to more generalized diets during the accretion of all rings past the eleventh ring (males = 0.58±0.05; females = 0.48±0.06), but this difference was only significant for male tortoises (two sample t-test; *P* = 0.006). Additionally, the mean individual trophic width (SEA_i_) for rings 1–11 (males = 2.3±0.2; females = 2.0±0.1) was less than the SEA_i_ for either males (3.2±0.3) or females (2.7±0.4) for growth periods after ring 11, and this difference was significant for male tortoises (two sample t-test; *P* = 0.017). The trophic width (SEA) for all males was smaller for rings grown before ring 11 compared to those grown after ring 11 (10.4±0.2 compared to 11.2±0.02; two sample t-test; *P* = 0.000), but for female tortoises the SEA was smaller in rings grown after ring 11 relative to those grown before (9.8±0.02 compared to 11.2±0.02; two sample t-test; *P* = 0.000). Male and female tortoises had similar mean SEA_i_s during growth periods before and after ring 11 (Table 5).

## Discussion

In general, desert tortoises are known to be highly individualistic specialized herbivores [19] and our analyses of the lifetime isotopic niche of this long-lived animal supports this observation (Tables 4 & 5; Figures 4a & 4b). Prior desert tortoise studies have painstakingly detailed the specific dietary preferences of individuals in a number of populations (e.g., [19], [46]). These studies have provided an immense amount of data that shows the specialized nature of tortoise diets over relatively short intervals of time (months to a few years). Here we have taken a different approach; we sacrifice the detail inherent in these earlier analyses for information that focuses on the ecosystem compartments (C_3_ versus C_4_/CAM plants) where tortoises obtain the nutrients and energy they need to fuel their entire life histories. We believe that is approach complements the specific dietary analyses and provides further insights into how a changing ecosystem might impact tortoise populations in the future.

In the following paragraphs, we examine the dietary niche of desert tortoise individuals and populations from several perspectives: 1) we discuss how total trophic niche breadth and diet variability differ among individuals and populations across a precipitation gradient, 2) we then examine how resource use varies among males and females, 3) we show how life history stage affects trophic niche breadth and diet variability in different resource environments, 4) we then compare insights from our research to those of the conventional dietary literature on desert tortoises. We note that metrics based on the standard ellipse area (SEA) result in lower estimates of dietary specialization relative to those based on the total area of convex polygons (TA), but here our focus is on the relative differences between populations and the two methods are in agreement in these comparisons.

### Trophic Niche Breadth and Dietary Variability Across a Precipitation Gradient

The desert mountain ranges inhabited by the desert tortoise in Arizona become successively drier as one moves from the eastern to the western part of the state due largely to less reliable and weaker summer rainfall experienced as the distance from the Mexican core of the North American Monsoon System increases in the more southwesterly portions of the Sonoran Desert [23]. The drier conditions and higher temperatures produce a decrease in the cover and diversity of C_3_, C_4_, and CAM plant species available to tortoises. This strong environmental gradient is reflected in the dietary choices made by tortoises; at the eastern and relatively productive wet end of the range (JAV), individual tortoises are highly specialized (SEA_i_/SEA_p_ = 0.35; TA_i_/TA_p_ = 0.13; Tables 4 & 5), while those living in the hottest and driest habitats (NW) to the west are more generalized (SEA_i_/SEA_p_ = 0.56; TA_i_/TA_p_ = 0.31; Tables 4 & 5). Additionally, the mean nearest neighbor distance (MNND; 0.20 compared to 0.41; Table 4) and the standard deviation of mean nearest neighbor distance (SDMNND; 0.19 compared to 0.50; Table 4) for tortoise growth rings in the driest populations were almost half as much as those in the wettest populations, indicating a higher degree of trophic overlap between and within individuals in drier environments.

Desert tortoises in the Sonoran Desert are most active during summer rains, with a smaller peak in activity during wet springs [38]. Tortoise activity also appears to be constrained by high air temperatures, and in one population activity was not observed at air temperatures above 40°C [47]. These observations suggest that in the hotter, more arid portion of their distribution, tortoises are likely to have shorter daily and seasonal activity periods and also must forage in a patchier, less vegetated landscape. One of the basic tenets of optimal foraging theory is that animals should minimize costs, and maximize intake while foraging. In the driest site, estimated ANPP is approximately 1/2 that of the most mesic site (46 g*m^−2^ versus 109 g*m^−2^), which would suggest that food plant encounter rates are lower. Consequently, tortoises should be less selective in their foraging choices. Conversely, desert tortoises in habitats with a higher plant diversity and abundance can afford to be more selective in their feeding choices and choose the most nutritionally superior plant resources, as desert tortoises are known to do when high quality resources are patchily distributed [18]. In short, optimal foraging theory states that when tortoises encounter profitable plant resources at a high rate, then the degree of diet specialization should be higher than when these same resources are encountered at lower rates [48]. Our observations are consistent with the above hypothesis and we found that as ANPP increased, population trophic niche width increased, and individual tortoises were more specialized in their feeding habits (Figures 4a & 4b; Tables 4 & 5). Tortoises living on the driest sites (e.g. ET and NW) occurred in a landscape of low C_3_, C_4_, and CAM plant species diversity and biomass which may equate to low resource encounter rates. Across the drier sites plant species diversity of all functional types, as well as the percent cover significantly declined. For example, the driest site (NW) had seven species of C_4_ forbs, 13 species of C_4_ grasses, and 70 species of C_3_ plants versus the 55 species of C_4_ forbs, 67 species of C_4_ grasses, and 465 species of C_3_ plants on one of the wettest sites (JAV; [49], [50]). As a consequence of this sparse and unreliable access to plant resources tortoises on these sites were dietary generalists situated within a reduced population-wide dietary niche (i.e., tortoises here cannot afford to be as selective in their foraging criteria; Tables 4 & 5). Individual tortoises on the drier sites had similar dietary niche breadths, but reduced population niche widths and less individual dietary specialization (Figure 4b; Tables 4 & 5) which may be explained by tortoises foraging on a lower diversity of plant resources (relative to wetter sites) whose availability is more unpredictable in these highly stochastic environments.

In contrast, desert tortoises inhabiting the more mesic sites (e.g., JAV) were exposed to higher resource levels and may have experienced higher resource encounter rates. They exhibited a greater degree of individual specialization and less between-individual trophic overlap across a more diverse resource base (Figure 4a). Individual tortoises thus occupied more divergent positions within the larger stable isotope niche space of more mesic habitats, and focused on a subset of the available stable isotope niche space. The observed decrease in dietary overlap between individual tortoises that accompanied the increased population niche width is notable because it provides support for the hypothesis that states larger population niche widths should be accompanied by a higher degree of between individual variation [6]; a hypothesis that has received some substantiation in other studies [4], [51], [52].

These more mesic sites also showed both a greater species diversity and greater preferred plant biomass that is likely to be available for more extended periods of time providing opportunities for specialization. Additionally, research on other populations of desert tortoises suggests that those tortoises found in areas of high precipitation have larger home ranges and make longer daily movements [53], [54]. This increased mobility is likely to increase their ability to encounter and graze preferred plant species in the patchily distributed resource landscape of the Sonoran Desert, increasing the tendency towards dietary specialization.

### Trophic Niche Breadth and Dietary Variability among Males, Females, and Juveniles

Female desert tortoises had growth rings depleted in δ^13^C relative to male tortoises, indicating higher use of C_4_/CAM plant resources. Additionally, the convex hull (TA_p_) area and standard ellipse area (SEA_p_) for male tortoises were larger than for female tortoises, but individual dietary specialization was similar for male and female tortoises (Tables 4 & 5). Seasonal patterns of activity are known to be different in male and female tortoises, which may affect seasonal patterns of foraging and plant use [55]–[57], [38], but whether the differences in SEA_p_ and TA_p_ between male and female tortoises have ecological or physiological ramifications remains ambiguous.

In this study we found that juvenile desert tortoises had a narrower dietary breadth and showed more dietary specialization relative to adult tortoises (Tables 4 & 5). Tortoise growth is most rapid through the first ten years of life [21], [58], [59] and slows as the animal nears reproductive maturity continuing at a slow rate towards asymptotic size [21], [59]. Our measurements of growth ring width in desert tortoises over the course of their lives showed that the period where growth rings 11 and 12 are laid down may represent a transition in the physiological ecology of individuals (Figure 5). Juvenile growth is rapid and ring widths are successively wider in individuals that have accrued fewer than 12 rings. Growth rings produced later in life (beyond ring 11) become progressively narrower as adult size is attained. During the early phase of rapid growth juvenile (i.e., the first growth rings accrued post-neonatal scute in adult tortoise growth ring series) male and female tortoises showed similarly narrow dietary breadth (mean female SEA_i_ = 2.0; mean male SEA_i_ = 2.3; Table 5) and dietary specialization (female SEA_i_/SEA_p_ = 0.37; male SEA_i_/SEA_p_ = 0.41; Table 5). Beyond ring 11 male and female tortoises showed a 40% and 35% expansion, respectively, in mean SEA_i_, and a 30% (females) and 41% (males) decrease in dietary specialization (Table 5). Conversely, the SEA_p_ for all females (but not males) was smaller for growth rings beyond ring 11 compared to rings 1 through 11. Though this may be a reflection of the potentially constrained foraging choices made by reproductive females, we lack the power to affirm this. These disparate patterns in plant resource use likely arise from the differences in the ecology and physiology of juvenile and adult tortoises. Juvenile desert tortoises, for example, show seasonal differences in patterns of activity and foraging compared to adult tortoises. Juvenile tortoises in the Mojave Desert are more active during winter and emerge from hibernation earlier in the spring than do larger tortoises [60], [61]. These observations suggest that juvenile Sonoran Desert tortoises are also able to take advantage of cooler conditions in late winter/early spring, when their preferred forbs have just emerged and their heights make them accessible to small tortoises. The physiology of juvenile tortoises also constrains the plant resources that they can eat, relative to those available to larger animals [62], [25]. The greater thermal inertia and mobility of large tortoises allow them to access a larger variety of patchily distributed plant species for a greater period of time. The reduced gut capacity and shorter retention times found in juvenile tortoises, as well as their smaller and weaker mandibles limits their foraging to relatively low-fiber, leafy C_3_ forbs whose availability may be temporally and spatially restricted [28], [19]. Although C_4_ grasses are a large component of the plant biomass produced in response to summer rain they are relatively inaccessible to very small tortoises due to handling constraints and digestive limitations. As tortoises grow larger, however, C_4_ grasses become an important part of their diet. Traditional observations of nutritional value suggest that C_3_ grasses should be favored over C_4_ grasses because of their higher nitrogen content, however, nutritional analyses of C_3_ and C_4_ grasses from southern Nevada show that actively growing C_4_ grasses contain significantly more nitrogen than C_3_ grasses found on the same sites [18].

### Comparing Traditional Diet Studies with Stable Isotope Approaches

Traditional diet studies provide detailed information on the specific plants from which tortoises feed by using bite count studies and fecal composition analyses [17], [18]. However, because tortoises are long-lived animals that balance nutrient, energy, and water budgets over months to years, these studies provide only a partial view of how desert tortoises interact with resources over their lifetimes. For long-lived animals such as tortoises it is important to complement these short-term detailed observations with studies that take a longer view and focus on resource compartments whose trajectories are subject to the directional effects of global warming. We thus estimated the proportion of the total lifetime diet that is harvested from these specific ecosystem compartments (C_3_ versus C_4_/CAM plants) across different tortoise populations. Because the growth of these different plant functional groups is tied to distinct and largely non-overlapping climatic conditions, we can begin to better understand how this consumer is coupled to its environment, as well as make predictions about how the desert tortoise will respond to changes in the availability of these plant functional groups. For example, the very detailed foraging observations made by Oftedal [46] on desert tortoises living on the MDF site reveal that tortoises feeding during the spring preferentially graze on C_3_ forbs (63% of 20,566 bites), particularly plants in the family Fabaceae, and that C_4_ forbs and C_4_ grasses make up a minor proportion of foraging effort (1% and 21% of bites, respectively). During the summer at this same site, however, tortoises preferentially select C_4_ grasses (70% of 39,949 bites), with a lesser interest in C_4_ forbs (18%). At this site CAM plant resources (cacti) make only a minor contribution to the desert tortoise’s energy and nutrient budget. The prickly pear cactus fruit (*Opuntia englemanni*), which is only available during the summer, was the only CAM resource used by tortoises at this site (4% of 39,949 bites). We also note that four of the eight sites (ST, MM, ET, and NW) do not have *Opuntia englemanni*, and the CAM plants that do occur on these sites are those that tortoises do not graze, such as cholla (*Cylindropuntia* sp.) and saguaro (*Carnegiea gigantea*). Our δ^13^C data show that different desert tortoise populations have a varying reliance on C_4_/CAM plant resources (from 14% on the MM site, to 39% on the MDF site), but that the majority of lifetime diet is harvested from the C_3_ plant ecosystem compartment across all sites. Although we are unable to identify particular species of C_3_ plants, or separate C_4_ plant versus CAM plant resource use on the basis of carbon isotope ratios alone, the work of Oftedal [46] and others indicates that C_3_ forbs, and especially plants in the genera *Lupinus*, *Lotus*, and *Astragalus* are likely to make up a major portion of the biomass ingested in the spring. Likewise, the observed patterns of C_4_/CAM resource use are largely due to the grazing of C_4_ grasses because cacti are only available on ½ of the sites and tortoises appear to show only very limited interest in cacti at sites where palatable cacti species are present.

### Conclusions

The information provided by our sampling approach, collecting dietary information that encompasses periods of 10 to 25+ years from many individuals, provides a pathway for examining how individual dietary specialization changes among populations occupying habitats along an environmental gradient. Here we uniquely use individual desert tortoises as ‘walking tree rings’. Through stable isotope analyses of growth ring chronologies we have the ability to easily examine the life-time dietary history of individual tortoises from many populations. We have demonstrated how individual consumers use resources across climatically variable habitats. The level of desert tortoise dietary specialization significantly declines in populations occupying hotter and drier landscapes with fewer plant resources. However, juvenile desert tortoises show consistently narrower dietary niche widths and a higher degree of dietary specialization. We provide insight into how a long-lived consumer responds to a variable environment, which is particularly relevant given the high importance of understanding and predicting how organisms will respond to projected climate changes. Desert tortoises in the Sonoran Desert, particularly those inhabiting very dry habitats, are nutritionally buffered by the bimodal ecosystem pulses of C_3_ and C_4_/CAM plant resources. In this system, a shift to a warmer and drier climate coupled with invasive C_4_ grass-fueled fire regimes may significantly alter the availability of the plant resource compartments required by desert tortoises to balance their energy and nutrient budgets, primarily through the reduced availability of C_3_ forbs and shrubs. This has the potential to negatively impact the growth and fitness of desert tortoises, particularly juveniles with a high degree of dietary specialization on C_3_ forbs.
